# BA6 Induces Apoptosis via Stimulation of Reactive Oxygen Species and Inhibition of Oxidative Phosphorylation in Human Lung Cancer Cells

**DOI:** 10.1155/2019/6342104

**Published:** 2019-05-07

**Authors:** Meng-Hsuan Cheng, Hung-Ling Huang, Yen-You Lin, Kuan-Hao Tsui, Pei-Chin Chen, Shu-Yu Cheng, Inn-Wen Chong, Ping-Jyun Sung, Ming-Hong Tai, Zhi-Hong Wen, Nan-Fu Chen, Hsiao-Mei Kuo

**Affiliations:** ^1^Division of Pulmonary and Critical Care Medicine, Department of Internal Medicine, Kaohsiung Medical University Hospital, Kaohsiung 80756, Taiwan; ^2^School of Medicine, College of Medicine, Kaohsiung Medical University, Kaohsiung 80708, Taiwan; ^3^Department of Respiratory Therapy, College of Medicine, Kaohsiung Medical University, Kaohsiung 80708, Taiwan; ^4^Department of Internal Medicine, Kaohsiung Municipal Ta-Tung Hospital, Kaohsiung Medical University Hospital, Kaohsiung 80145, Taiwan; ^5^Department of Orthopaedic Surgery, Ping-Tung Christian Hospital, Pingtung 90059, Taiwan; ^6^Department of Obstetrics and Gynecology, Kaohsiung Veterans General Hospital, Kaohsiung 81362, Taiwan; ^7^Department of Obstetrics and Gynecology and Institute of Clinical Medicine, National Yang-Ming University, Taipei 11221, Taiwan; ^8^Department of Marine Biotechnology and Resources, National Sun Yat-sen University, Kaohsiung 80424, Taiwan; ^9^Doctoral Degree Program in Marine Biotechnology, National Sun Yat-sen University, Kaohsiung 80424, Taiwan; ^10^Graduate Institute of Marine Biology, National Dong Hwa University, Pingtung 944, Taiwan; ^11^National Museum of Marine Biology and Aquarium, Pingtung 944, Taiwan; ^12^Institute of Biomedical Sciences, National Sun Yat-sen University, Kaohsiung 80424, Taiwan; ^13^Center for Neuroscience, National Sun Yat-sen University, Kaohsiung 80424, Taiwan; ^14^Department of Neurosurgery and Surgery, Kaohsiung Armed Forces General Hospital, Kaohsiung 80284, Taiwan; ^15^Department of Neurological Surgery, Tri-Service General Hospital, National Defense Medical Center, Taipei 11490, Taiwan

## Abstract

Lung cancer is the leading cause of cancer deaths in the world, with a five-year survival rate of less than 30%. Clinically effective chemotherapeutic treatments at the initial stage may eventually face the dilemma of no drug being effective due to drug resistance; therefore, finding new effective drugs for lung cancer treatment is a necessary and important issue. Compounds capable of further increasing the oxidative stress of cancer cells are considered to have anticancer potential because they possessed the ability to induce apoptosis. This study mainly investigated the effects of BA6 (heteronemin), the marine sponge sesterterpene, on lung cancer cell apoptosis, via modulation of mitochondrial reactive oxygen species (mtROS) and oxidative phosphorylation (OXPHOS). BA6 has cellular cytotoxic activities against a variety of cancer cell lines, but it has no effect on nontumor cells. The BA6-treated lung cancer cells show a significant increase in both cellular ROS and mtROS, which in turn caused the loss of mitochondrial membrane potential (MMP). The increase of oxidative stress in lung cancer cells treated with BA6 was accompanied by a decrease in the expression of antioxidant enzymes Cu/Zn SOD, MnSOD, and catalase. In addition, OXPHOS performed in the mitochondria and glycolysis in the cytoplasm were inhibited, which subsequently reduced downstream ATP production. Pretreatment with mitochondria-targeted antioxidant MitoTEMPO reduced BA6-induced apoptosis through the mitochondria-dependent apoptotic pathway, which was accompanied by increased cell viability, decreased mtROS, enhanced MMP, and suppressed expression of cleaved caspase-3 and caspase-9 proteins. In conclusion, the results of this study clarify the mechanism of BA6-induced apoptosis in lung cancer cells via the mitochondrial apoptotic pathway, suggesting that it is a potentially innovative alternative to the treatment of human lung cancer.

## 1. Introduction

Lung cancer is the most common type of cancer diagnosed in the world and is the leading cause of death of most American cancer patients. According to estimates, there will be about 234,000 new lung cancer patients in the United States in 2018 and about 154,000 patients will die of lung cancer [1]. In Taiwan, lung cancer is also the leading cause of cancer deaths, with a 5-year survival rate of less than 30% [2]. As the initial symptoms of lung cancer are not obvious, and there is no effective screening tool, about 50% of patients will die within one year after being diagnosed. Even though the cost of treating lung cancer in the United States is as high as USD 10.3 billion per year, it cannot reduce the above-mentioned high mortality rate [3]. Around 15% of Caucasian and 40% of Asian non-small-cell lung cancer patients with epidermal growth factor receptor (EGFR) mutation can be treated with targeted therapy; thus, the average survival of this patient group can be 24 to 30 months; however, most patients with advanced lung cancer receiving the standard platinum-based combination chemotherapy only survive about 12 months on average [4]. In addition, regardless of whether the targeted drug can be used, the patient will eventually face the difficulty of no available drug and will die; thus, the development of new drugs for lung cancer treatment remains an urgent issue.

Apoptosis, also known as programmed cell death, is mainly caused by the extrinsic pathway, the intrinsic pathway, and endoplasmic reticulum stress (ER stress) [5]. The intrinsic pathway is mainly via the participation of caspases (cysteinyl aspirate-specific proteases). Caspase is a very important medium in the process of apoptosis and is usually present in the cytoplasm in an inactive form; thus, it must be hydrolyzed by proteases to the active form before the work of apoptosis can be carried out [6]. The caspase-dependent intrinsic pathway is regulated by a group of proteins on the mitochondrial membrane and cytoplasm. Reactive oxygen species (ROS) in cells are mainly produced by oxidative phosphorylation (OXPHOS) in mitochondria. In many human diseases, such as cancer and neurodegenerative diseases, changes of ROS can be observed [7–9]. The OXPHOS mechanism mainly provides the source of cellular energy and drives all reactions in the cells, which consists of the electron transport chain (ETC) and ATP synthase. Regarding the measurement of the mitochondrial respiratory function, using extracellular flux analysis is the best method for detecting intact cells to measure basal respiration, ATP production, proton leak, maximal respiration, spare respiratory capacity, and nonmitochondrial respiration, which provides a basis for further study of mitochondrial functions and bioenergetics [10]. When cells are under stress and injury, such as radioactive radiance, hypoxia, drugs, and DNA damage, they will produce ROS. Excessive ROS will destroy the mitochondria, cause a decrease in mitochondrial membrane potential (MMP), impair ATP production, and ultimately result in mitochondrial dysfunction. Furthermore, release of cytochrome C from damaged mitochondria plays a central role in the execution phase of apoptosis [8, 10, 11].

BA6, also known as heteronemin, is a marine sesterterpene isolated from *sponge*, and its chemical molecular formula is C_29_H_44_O_6_. It has been proven to cause apoptosis and autophagy in human renal carcinoma cells and leukemia cells [12, 13]. The functions of the distal organs are impaired in most cancer patients due to distant metastasized cancer cells. As BA6 can inhibit the intravasation of the initial breast cancer cells, it is capable of avoiding the potential of distal metastasis [14]. To date, BA6 has not been discussed in depth in the treatment of lung cancer; thus, the mechanism is unclear. This paper mainly elucidates the effect of B6 on the mitochondrial function and oxidative stress of lung cancer cells.

## 2. Materials and Methods

### 2.1. Cell Lines and Cell Culture

This study used several cells, including human lung carcinoma cells (A549 cells—ATCC® CCL-185™), human hepatoma cells (HepG2—ATCC® HTB-8065™), and two human glioblastoma cell lines (GBM-8401 (purchased from the Food Industry Research and Development Institute, Taiwan) and U87 (ATCC® HTB-14™)). Human primary gingival fibroblast (HGF-ATCC® PCS-201-018™) and oral mucosal fibroblast (OMF) were from Professor Michael Hsiao at the Academia Sinica Institute. A549 cells were maintained in the F12K medium (Gibco, Darmstadt, Germany) with L-glutamine. HepG2, GBM, U87, HGF, and OMF cells were cultured with Minimum Essential Eagle's medium (Gibco). These cell lines were cultured with 10% heat-inactivated fetal calf serum (FCS; Invitrogen, Carlsbad, CA, USA) and penicillin/streptomycin in 5% CO_2_/95% O_2_ humidified incubator at 37°C.

### 2.2. Cell Treatment and Reagent

A sliced body of the sponge *Hyrtios erecta* (wet and dry weights, 900.0 and 164.0 g, respectively) was extracted with a mixture of organic solvent (MeOH : CH_2_Cl_2_ = 1 : 1; volume ratio). The resulting 38.3 g extract was partitioned with EtOAc and H_2_O. The EtOAc layer (5.3 g) was separated on silica gel and eluted with a mixture of n-hexane/EtOAc (stepwise from 100 : 1 to 100% EtOAc; volume ratio) to yield 37 fractions. Fraction 11 (1.6 g) was separated by silica gel column chromatography and then eluted with a mixture of n-hexane/EtOAc (3 : 1) to afford BA6 (386.1 mg) [15]. Stock solution BA6 was prepared in DMSO and stored at −20°C. Cells were treated with the indicated concentrations of BA6 for 24 h. Antibodies against caspase-3, caspase-9, Bax, Bcl-2, and cytochrome C were purchased from Cell Signaling Technology (Danvers, MA, USA). Anti-COX IV, anti-*β*-actin, anti-Cu/ZnSOD, anti-MnSOD, and anti-catalase antibodies were obtained from Santa Cruz Biotechnology (CA, USA). MitoTEMPO obtained from Sigma-Aldrich (St. Louis, MO, USA) was a mitochondria-targeted antioxidant and a specific scavenger of mitochondrial superoxide. In order to determine the importance of mitochondrial ROS of BA6 treatment, A549 cells were pretreated with or without MitoTEMPO for 4 h before harvest for further analysis. The Annexin V/PI kit was purchased from Molecular Probes (Carlsbad, CA, USA). The In Situ Cell Death Detection Kit for the detection and quantification of apoptosis at the single-cell level, labeling of DNA strand breaks (TUNEL technology), was purchased from Roche Applied Science (Mannheim, Germany). 3-(4,5-Dimethylthiazol-2-yl)- 2,5-diphenyltetrazolium bromide (MTT), MitoSOX Red, JC-1, rhodamine 123, and DCFH-DA were purchased from Thermo Fisher Scientific and dissolved in DMSO or PBS.

### 2.3. MTT Assay for Cell Viability

The MTT assay was performed according to the manufacturer's instructions. Briefly, cells were seeded at an initial density of 1 × 10^4^ per well in 96-well plates and incubated overnight before being treated with or without MitoTEMPO for 4 h and then incubated with various concentrations of BA6 for 24 h. Cells were then observed under a microscope (Leica Microsystems, Wetzlar, Germany), and the images were captured with a SPOT CCD RT-slider integrating camera (Diagnostic Instruments, Sterling Heights, MI, USA). The treated cells were subsequently incubated in a medium containing MTT (0.5 mg/ml) for an additional 3 h. After removal of the supernatant, 50 *μ*l DMSO was added to each well to dissolve the intracellular formazan compound. The absorbance value at a wavelength of 570 nm was measured by an ELISA reader (Dynatech Laboratories, Chantilly, VA, USA). The relative cell viability (%) was calculated as the percentage between BA6-treated cells and the untreated controls, expressed as mean ± SEM. IC_50_ values were obtained from CalcuSyn software (Biosoft, Ferguson, MO, USA), and the values are the mean of three independent experiments.

### 2.4. Apoptosis Detection

#### 2.4.1. Annexin V/PI Stain

The annexin V/PI stain was used to detect late apoptosis and early apoptosis, as induced by BA6. 5 × 10^5^ A549 cells were plated in 6-well plates and incubated overnight. A549 cells were treated with or without MitoTEMPO for 4 h, and BA6 indicated concentrations of 0.01, 0.1, 1, and 10 *μ*M, respectively, while untreated cells served as the control (0 *μ*M). The resuspended cells at 1 × 10^5^ cells/100 *μ*l in 1X binding buffer contain 5 *μ*l purified recombinant annexin V and 5 *μ*l PI. The cells were gently mixed and incubated for 15 min at RT to avoid light, and 500 *μ*l of 1X binding buffer was added. At least 20,000 events were acquired and analyzed from each sample by flow cytometry (CytoFLEX, Beckman).

#### 2.4.2. Living Cell Tomographic Microscopy Images

A549 cells were seeded overnight in a glass bottom microwell dish after BA6 treatment with concentrations of 0 (as control) and 10 *μ*M for 24 h. However, visualization of the 3D live cell morphology of A549 cells and localization of QD were performed by interferometric detection using a live cell tomographic holographic 3D microscope Nanolive (3D Cell Explorer, Lausanne, Switzerland) and processed using STEVE software (3D Cell Explorer). This study took a photo every 3 minutes, shooting time is for 3 hours, and the images were recorded.

#### 2.4.3. Terminal Deoxynucleotidyl Transferase- (TdT-) Mediated dUTP Nick End-Labeling (TUNEL)

TUNEL was used to detect late apoptosis, as induced by BA6. Briefly, A549 cells were seeded in a glass bottom microwell dish for 24 h, after BA6 treatment with concentrations of 0 (as control) and 10 *μ*M for 24 h. The culture medium was removed; the cells were washed twice with PBS and fixed in 4% paraformaldehyde for 10 min at 4°C. TUNEL analysis was performed using an In Situ Cell Death Detection Kit Fluorescein according to the instructions. TUNEL-positive cells were visualized by immunofluorescent microscopy (Leica Microsystems; Wetzlar, Germany). TUNEL-positive cells containing fluorescence were identified by counterstaining with DAPI for 10 min at RT. The formation of a greenish fluorescent set at 480 nm and bluish fluorescent set at 405 nm was observed on the slides, and the images were captured with a SPOT CCD RT-slider integrating camera (Diagnostic Instruments, Sterling Heights, MI, USA). TUNEL-stained apoptosis cells were observed with filters for green fluorescence, and blue indicated the cell nucleus.

### 2.5. Flow Cytometry Analysis for ROS and Membrane Potential

The intracellular ROS (hydrogen peroxide) level was detected by 2′, 7′-dichlorofluorescin diacetate (DCFH-DA). Mitochondria-specific ROS measuring dye (MitoSOX Red) was used to detect mtROS by flow cytometry. Rhodamine 123 is a mitochondria-specific fluorescent cationic dye, which is used to monitor mitochondrial membrane potential activation under BA6 drug treatment. Mitochondrial membrane depolarization was detected by utilizing fluorescent dye JC-1. The cells were seeded at a concentration of 5 × 10^5^ cells in each well in a 6-well plate. After treatment with various BA6 drugs or MitoTEMPO, cotreat with BA6 and MitoTEMPO, respectively, four fluorescent dyes were added at proper concentrations in the HBSS solution, which were incubated for suitable times at 37°C. Subsequently, on the removed media, the cells were trypsinized and resuspended in 1 ml PBS. Beckman Coulter's CytoFLEX flow cytometry (Southfield, MI, USA) was used to detect the fluorescence intensity of intracellular ROS, mtROS, mitochondrial membrane potential, and JC-1. At least twenty thousand cells per group were analyzed using the flow cytometry analysis Beckman software CytExpert 2.0.

### 2.6. Mitochondrial Function Measurements

**Figure 1:**
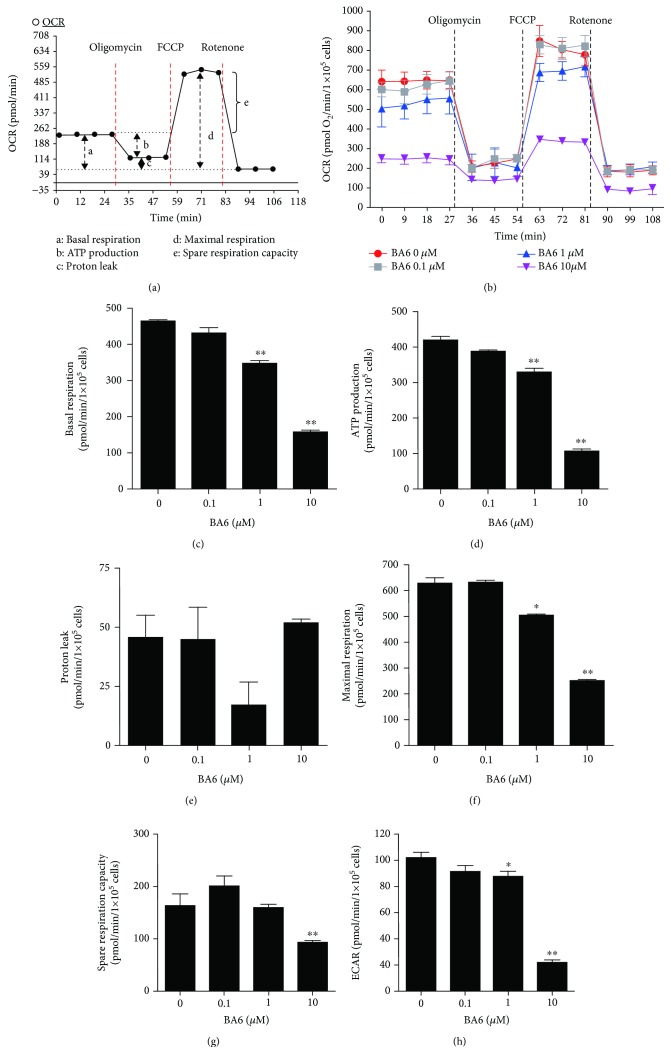
Effect of various concentration BA6 on mitochondrial function in A549 cells. (a) The mitochondrial respiration assay started with four basal OCR measurements, followed by continuous injections of oligomycin, FCCP, and rotenone. The basic parameters measured include basal respiration, ATP production, proton leak, maximum respiration, and spare respiratory capacity. (b) Time course and OCR curve plot. OCR in basal and BA6 treatment conditions (0.1, 1, and 10 *μ*M for 24 h) were examined by the Seahorse XF24 analyzer in the absence or presence of oligomycin (1 *μ*M), FCCP (0.25 *μ*M), and rotenone (1 *μ*M). Quantification of changes in parameters induced by BA6 treatment includes the basal respiration (c), ATP production (d), proton leak (e), maximum respiration (f), spare respiratory capacity (g), and ECAR (i). OCR and ECAR levels are quantified by normalization of cell numbers. Data were obtained from three independent experiments and expressed as the mean ± SEM. ^∗^ *p* < 0.05 and ^∗∗^ *p* < 0.01, as compared with untreated cells.

The oxygen consumption rate (OCR) and extracellular acidification rate (ECAR) in cells and mitochondria were determined using a Seahorse XF24 Extracellular Flux Analyzer (Seahorse Bioscience Inc., Chicopee, MA, USA). For comparison between experiments, the data are expressed as OCR of pmol/min/1 × 10^5^ cells and ECAR of mpH/min/1 × 10^5^ cells. In the beginning, 1 × 10^5^ cells were seeded on Seahorse XF24 Cell Culture Microplates. After overnight incubation, cells were treated with 0, 0.01, 0.1, 1, and 10 *μ*M BA6 for 24 h. After washing the cells with 0.5 ml of DMEM without sodium bicarbonate, pH = 7.4, and then, 675 *μ*l of DMEM was added to each well for further examination. The base OCR was measured four times, plotted as a function of the cells under basal conditions, and then, 1 *μ*M oligomycin, 0.25 *μ*M carbonyl cyanide-4-(trifluoromethoxy) phenylhydrazone (FCCP), and 1 *μ*M rotenone were added sequentially as indicated (Figure 1(a)). At the end of recording, cells were collected and counted by using a trypan blue exclusion assay, and then, the OCR and ECAR values were calculated after normalization with the number of cells.

### 2.7. Mitochondrial and Cytosol Isolation Methods

The A549 cells were treated with various concentrations of BA6; the mitochondria and cytosol separation in the A549 cells was isolated according to the manufacturing protocol of the Mitochondria/Cytosol Fractionation kit (BioVision Inc., Milpitas, CA, USA), and then, according to the “Western blot analysis” method, the mitochondrial or cytoplasmic cytochrome C was measured.

### 2.8. Western Blot Analysis

After pretreatment with or without 10 *μ*M MitoTEMPO for 4 h, A549 cells were cultured with 0, 0.01, 0.1, 1, and 10 *μ*M BA6 for 24 h. Cells were then harvested and incubated in a protein extraction reagent (Thermo Fisher Scientific, USA), and then, the lysates were centrifuged at 13,000 rpm at 4°C for 30 min to obtain supernatant soluble proteins. The BCA (Bio-Rad, Hercules, CA, USA) assay was used to determine protein concentrations. The extracted proteins were loaded onto SDS-PAGE, and separated proteins were transferred to PVDF membranes (Millipore, Bedford, MA, USA). The membrane was blocked with 5% milk and then incubated overnight at 4°C with primary antibodies. The membranes were incubated at 37°C for 1 h with HRP-conjugated 2nd antibodies and then placed on a visualization strip using a chemiluminescence kit (Millipore, Darmstadt, Germany) detection membrane and UVP BioChemi imaging (UVP LLC, Upland, CA, USA). The relative densitometry of the bands was quantified using LabWorks 4.0 software (UVP LLC). The PVDF membrane was reprobed using a *β*-actin antibody as a loading control.

### 2.9. ATP Concentration

A549 cells were seeded in triplicate at a density of 2 × 10^5^ cells/well in 6-well plates and treated with various concentrations of BA6 for 24 h. The cells were harvested in a lysis buffer (containing 20 mM glycine, 50 mM MgSO_4_, and 4 mM EDTA). ATP measurements were carried out according to the manufacturer's instruction with an ATP Colorimetric/Fluorometric assay kit (BioVision Inc., Milpitas, CA, USA). Twenty microliters (20 *μ*l) from each sample was mixed with 4 *μ*l ATP solution for fluorescence readings at Ex/Em 535/587 nm using a fluorescence meter. The amount of ATP production was determined from a standard curve constructed with 10–100 pmol ATP.

### 2.10. Statistical Analysis of Studies

A Social Science program (SPSS for Windows, version 17; SPSS Inc., Chicago) for Windows 13.0 was used for all statistical analysis data. The variables analyzed by an independent *t*-test are presented as mean ± standard error (SEM). According to the results, as analyzed by the *t*-test, ^∗∗^ *p* < 0.01 or ^∗^ *p* < 0.05 was considered to be statistically significant.

## 3. Results

### 3.1. Cytotoxicity of BA6 in Different Cancer Cell Lines

**Figure 2:**
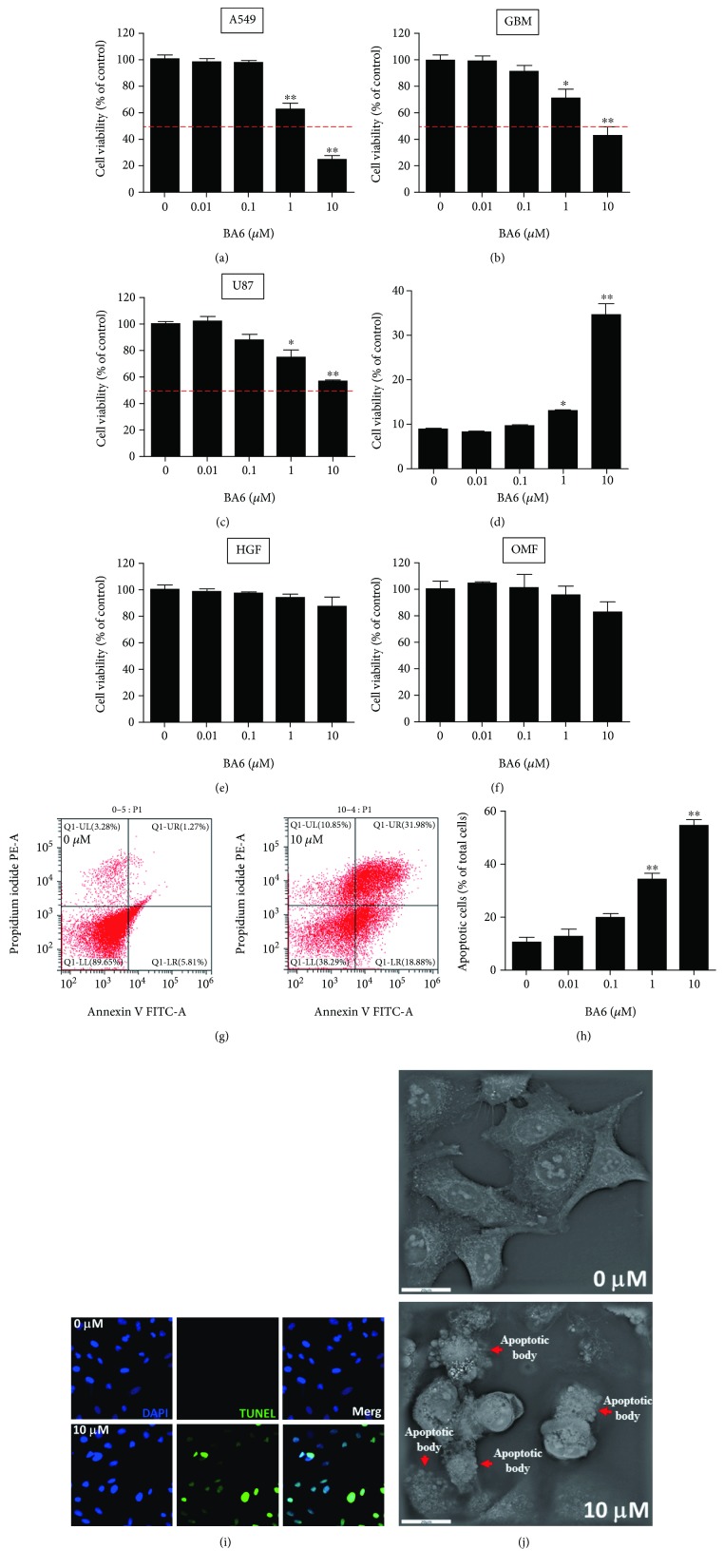
Effect of BA6 on the viability of various cancer cells and the induction of apoptosis in A549 cells. BA6 reduced the viability of various cancer cells. A549 cells (a), GBM cells (b), U87 cells (c), HepG2 cells (d), HGF cells (e), and OMF cells (f) were treated with the 0, 0.01, 0.1, 1, and 10 *μ*M concentrations of BA6 for 24 h, respectively, and then, an MTT assay was performed to measure cell viability. Cell viability (%) was expressed as a percentage, as compared to the untreated cells (0 *μ*M). The results are expressed as the mean ± SEM of three independent experiments. The apoptosis assay of BA6-treated A549 cells was detected by annexin V-FITC (green fluorescence)/propidium iodide (red fluorescence) staining flow cytometry and immunofluorescence TUNEL (green fluorescence) staining. (g) A549 cells treated without and with BA6 (10 *μ*M) for 24 h are shown with representative four quadrant dot plots from FITC-conjugated annexin V and PI staining. (h) The percentages of early apoptosis (lower right quadrant) and late apoptosis (upper right quadrant) from flow cytometric analysis for A549 cells treated with BA6 for 24 h were assessed. The apoptotic A549 cells were increased, along with increasing concentrations of BA6. Total cells are 20,000 events, and values are the mean ± SEM of three independent experiments. (i) Immunofluorescence showed apoptotic A549 cells marked by the TUNEL assay without or with BA6 (10 *μ*M) treatment for 24 h. The cell DNA/nuclei were stained using DAPI (blue color) and visualized under a laser confocal microscope (400x). (j) Living cell tomographic microscopy images of A549 cells untreated or treated with BA6 (10 *μ*M). The significance was determined by Student's *t*-test: ^∗^ *p* < 0.05 and ^∗∗^ *p* < 0.01, as compared with the control group.

We first determined cell viability under BA6 treatment on cancer and nontumor cells. According to our results, the cytotoxicity of BA6 treated for 24 h in various cancer cell lines showed a concentration-dependent manner by the MTT stain method. At BA6 concentrations of 1 and 10 *μ*M, A549 cell viability was significantly decreased to 62.3 ± 4.7% and 24.3 ± 2.8% of the control (100 ± 3.1%) level, respectively (Figure 2(a)). At BA6 concentrations of 1 and 10 *μ*M, GBM cell viability was significantly reduced to 71.4 ± 6.5% and 43.3 ± 6.5% of the control (100 ± 3.9%) level, respectively (Figure 2(b)), and U87 cell viability significantly reduced to 74.7 ± 5.3% and 56.6 ± 0.9% of the control (100 ± 1.6%) level, respectively (Figure 2(c)). At BA6 concentration of 10 *μ*M, HepG2 cell viability was significantly reduced to 60.8 ± 1.1% of the control (100 ± 0.9%) level, respectively (Figure 2(d)). However, BA6 did not reveal obvious cytotoxic effect on nontumor cells, including HGF-1 cells (Figure 2(e)) and OMF cells (Figure 2(f)). These results suggest that, while BA6 can inhibit the cell viability of the cancer cells, there is no influence on nontumor cells.

### 3.2. Effect of Apoptosis by BA6 in A549 Cells

Our preliminary results showed that the cytotoxic effect of BA6 was more significant in A549 cells. The annexin V/propidium iodide (PI) double staining and flow cytometry analysis were used to detect apoptosis in BA6-treated A549 cells.  Figure 2(g) indicates that annexin V-/PI- shows survival cells (left, down), annexin V+/PI- means early apoptotic cells (right, down), and the distribution of annexin V+/PI+ represents late apoptotic cells (right, up). Apoptotic cells, including early and late apoptosis, increased from 10.3 ± 1.9% in the untreated group to 34.0 ± 2.2% and 54.1 ± 2.2% in the treatment 1 and 10 *μ*M BA6 groups (Figures 2(g) and 2(h)). The TUNEL assay can detect DNA strand breaks that are mainly induced by apoptosis. TUNEL and DAPI staining were used to confirm BA6-induced apoptosis and DNA fragmentation in A549 cells, respectively. By means of confocal microscopy observation, the cells treated with BA6 showed a strong green fluorescence (TUNEL) and a blue fluorescence (DAPI-nuclear indication position), which indicated DNA fragmentation and chromatin condensation (Figure 2(i)). BA6 was treated in A549 cells for 24 h and photographed in a tomographic holographic 3D microscope. We observed that many apoptotic bodies appeared in the BA6-treated group (Figure 2(j)). More detailed imaging of BA6 treatment is shown in Supplemental movie 01 for 3 h. Therefore, we confirmed that marine natural extract BA6 can lead to changes in the structure and content of the nuclear DNA and induce apoptosis in A549 cells. Taken together, the results from annexin/PI, TUNEL stain, and the 3D cell morphologies show that BA6 treatment significantly enhanced apoptosis in A549 cells.

### 3.3. Effect of BA6 on the ROS Production and Mitochondrial Membrane Potential (MMP)

**Figure 3:**
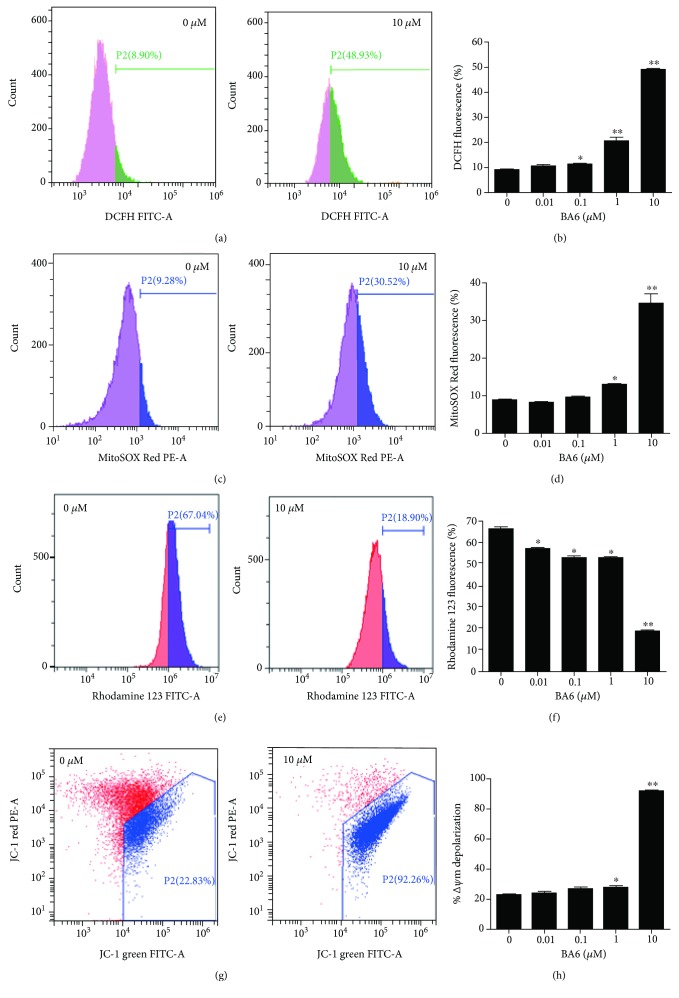
BA6 induced intracellular and mitochondrial ROS production and dissipation of MMP in A549 cells. (a) The probe of DCFH-DA (green) fluorescence was used to detect intracellular ROS (shifted towards the right) by flow cytometry in A549 cells treated without or with 10 *μ*M BA6 for 24 h. (b) Relative quantification of BA6-induced cellular ROS production. Values are the mean ± SEM of three independent experiments. (c) The probe of MitoSOX Red (red) fluorescence was used to detect mtROS (shifted towards the right) by flow cytometry in A549 cells treated without or with 10 *μ*M BA6 for 24 h. (d) Relative quantification of BA6-induced mtROS production. Values are the mean ± SEM of three independent experiments. (e) The mitochondrial membrane potential and depolarization were monitored by fluorescence dye of rhodamine 123 and JC-1 in A549 cells. Rhodamine 123 (green fluorescence) dyes were used to detect the membrane potential of the mitochondria. The histogram showed rhodamine 123 signals from untreated or treated 10 *μ*M BA6 for 24 h, and the left shift represents the loss of MMP. (f) Relative quantification of BA6-induced disruption of MMP. The percentages of cells with intact MMP were decreased, along with increasing concentration of BA6. (g) The aggregate fluorescent count was indicative of mitochondrial depolarization by JC-1 staining and flow analysis assessment. JC-1 can be discriminated by a set gating measurement area. The presentative flow cytometry dot plots demonstrated A549 cells treated without or with 10 *μ*M BA6 for 24 h. (h) Quantification analysis of the percentages of cells with depolarized mitochondria after treatment indicated concentrations of BA6 for 24 h. All total cells are 20,000 events, and the results are presented as the mean ± SEM of three independent experiments. ^∗^ *p* < 0.05 and ^∗∗^ *p* < 0.01 vs. the untreated BA6 (0 *μ*M) group.

Mitochondria are the main locations of ROS production. The cellular and mitochondrial ROS could be separately detected by flow cytometry using DCFH and a MitoSOX Red probe. The flow cytometry histogram showed that treatment with the BA6 marine drug led to increased cellular ROS in A549 cells (Figure 3(a)). With 1 and 10 *μ*M doses of BA6 treatment for 24 h, the cellular ROS rates were significantly increased to 20.6 ± 1.5% and 49.0 ± 0.4% of the control (9.0 ± 0.3%) level, respectively (Figure 3(b)). The flow histogram showed that treatment with BA6 increased mtROS in A549 cells (Figure 3(c)), and at concentrations of 1 and 10 *μ*M doses of BA6, the mtROS was significantly increased to 13.0 ± 0.1% and 34.4 ± 2.6% of the control (8.9 ± 0.3%) level, respectively (Figure 3(d)). According to abundant research documents, the collapse of the mitochondrial membrane potential occurs in different types of cells and tissues when suffering from excessive oxidative stress by ROS overproduction [16]. MMP was detected by flow cytometry using rhodamine 123 and JC-1 staining. The flow histogram plots after rhodamine 123 staining showed that treatment with BA6 led to a decrease of MMP in A549 cells (Figure 3(e)). Treatment with BA6 (0.01, 0.1, 1, and 10 *μ*M) significantly decreased MMP to 57.2 ± 0.6%, 53.0 ± 1.0%, 53.0 ± 0.5%, and 19.1 ± 0.1% of the control (66.6 ± 0.6%) level, respectively (Figure 3(f)). In JC-1 staining, a heavy shift of A549 cells from the left upper quadrant towards the right lower quadrant was detected in 10 *μ*M BA6 treatment, and the data showed that treatment with BA6 resulted in lower MMP (Figure 3(g)). Treatment with 1 and 10 *μ*M BA6 significantly increased the cell population with mitochondrial depolarization to 28.0 ± 1.3% and 92.1 ± 0.3% of the control (23.0 ± 0.6%) level, respectively (Figure 3(h)). In summary, these results suggest that BA6 induces both cellular ROS and mtROS productions as well as MMP disruption, leading to A549 cell apoptosis.

### 3.4. BA6 Inhibits Oxygen Consumption Rate and Extracellular Acidification Rate of Human A549 Cells

As shown in Figure 1(a), the several mitochondrial respiration stages were calculated based on the oxygen consumption rate (OCR) after the sequential addition of the respiration inhibitors of oligomycin, FCCP, and rotenone to inhibit the electron transport chain. The OCR and detection time curve plots show that treatment with BA6 led to decreased OCR in A549 cells. The base OCR was measured four times and plotted as a function of the cells under basal conditions, and then, 1 *μ*M oligomycin, 0.25 *μ*M FCCP, and 1 *μ*M rotenone were added as indicated. Various concentrations of BA6 were added to detect the changes of OCR (Figure 1(b)). The basal respiration OCR values decreased to 347 ± 8.2 (1 *μ*M) and 157.3 ± 6.2 pmol/min/1 × 10^5^ cells (10 *μ*M), respectively, as compared to the control (465 ± 3.2 pmol/min/1 × 10^5^ cells), which showed a dose-dependent manner (Figure 1(c)). The couple respiration (ATP production) value was attenuated to 329.7 ± 9.8 (1 *μ*M) and 106.3 ± 5.8 pmol/min/1 × 10^5^ cells (10 *μ*M), as compared with the control 419.7 ± 10.9 pmol/min/1 × 10^5^ cells (Figure 1(d)). The trend of proton leak (uncouple respiration) did not show significant difference (Figure 1(e)). The separate addition of 1 and 10 *μ*M BA6 significantly reduced the maximal respiration of 505.3 ± 5.4 and 250 ± 5.6 pmol/min/1 × 10^5^ cells, as compared with the control 628.0 ± 23.1 pmol/min/1 × 10^5^ cells (Figure 1(f)). Spare respiration capacity decreased to 92.3 ± 4.7 pmol/min/1 × 10^5^ cells after 10 *μ*M BA6 treatment, as compared to the control 163.0 ± 22.3 pmol/min/1 × 10^5^ cells (Figure 1(g)). Nonmitochondrial respiration-ECAR value was decreased to 87.3 ± 4.3 mpH/min/1 × 10^5^ cells (1 *μ*M BA6) and 21.7 ± 2.2 mpH/min/1 × 10^5^ cells (10 *μ*M BA6), respectively, as compared to the control 101.9 ± 4.3 mpH/min/1 × 10^5^ cells (Figure 1(h)). These results suggest that the basal respiration, ATP production, maximal respiration, spare capacity, and ECAR were significantly inhibited after BA6 treatment in A549 cells, while the proton leak remained unaffected.

### 3.5. Effect of BA6 on the Antioxidants and Mitochondrial Energy Production

**Figure 4:**
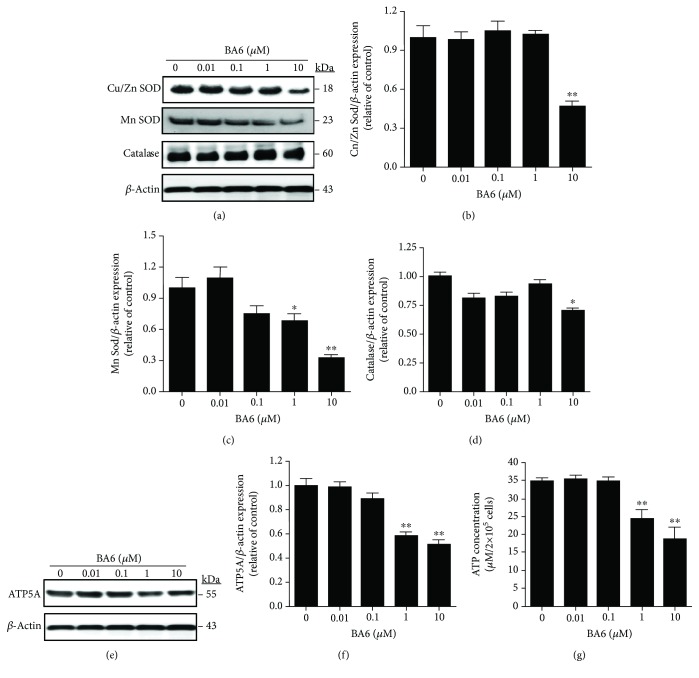
Effect of BA6 on the changes of the metabolizing enzymes of reactive oxygen species (ROS) and energy metabolism in A549 cells. (a) A549 cells were treated with various concentrations of BA6 for 24 h. Cell lysates were loaded for Western blot analysis using various antibodies, including Cu/Zn SOD, MnSOD, catalase, and *β*-actin antibodies. *β*-Actin was detected as a normalization internal control. The Cu/Zn SOD (b), MnSOD (c), and catalase (d) protein levels were quantified by ImageJ software and normalized with that of the *β*-actin level. (e) Western blot showed the changes of the protein level of ATP5A in BA6-treated A549 cells. The *β*-actin level was used as the loading control. (f) The ATP5A protein band was quantified by ImageJ software and normalized with that of the *β*-actin level. (g) The effects of various doses of BA6 treatment for 24 h on the total ATP production. The values were calculated as mean ± SEM from three independent experiments. Significance was determined by *t*-test: ^∗^ *p* < 0.05 and ^∗∗^ *p* < 0.01 as compared with untreated (0 *μ*M) cells.

The superoxide dismutase family, meaning Mn superoxide dismutase (MnSOD), is located in the mitochondrial matrix, while Cu/Zn superoxide dismutase (Cu/Zn SOD) is found in the mitochondrial intermembrane, cytoplasm, and extracellular area, which protects cells against excessive oxidative stress. These enzymes can extensively catalyze undue ROS in both the mitochondria and cytoplasm in a variety of cell types and therefore were involved in ROS production and changes of the respiratory chain complex process [17]. We next examined whether the antioxidants were involved in BA6-induced ROS overproduction in A549 cells, and the results revealed that BA6 treatment downregulated the protein expression of Cu/Zn SOD, MnSOD, and catalase (Figure 4(a)). The expression of Cu/Zn SOD was significantly downregulated after 10 *μ*M BA6 treatment (Figure 4(b)). The 10 *μ*M BA6 treatment decreased the protein expressions of MnSOD by 65% and catalase by 30% (Figures 4(c) and 4(d)). To determine if BA6 can affect mitochondrial energy metabolism, we further detected the changes of the ATP5A protein expression after BA6 incubation.  Figure 4(e) shows that the ATP5A protein level was inhibited in a dose-dependent manner and was decreased by 50% after 10 *μ*M BA6 treatment for 24 h (Figure 4(f)). In line with protein expression, the ATP production was extensively inhibited from 34.7 ± 0.9 *μ*M/2 × 10^5^ cells (control group) to 24.4 ± 2.5 *μ*M/2 × 10^5^ cells and 18.7 ± 3.2 *μ*M/2 × 10^5^ cells in 1 and 10 *μ*M BA6-treated group, respectively (Figure 4(g)). These results indicate that BA6 can potently induce excessive oxidative stress through the inhibition of antioxidants, leading to a decrease of ATP production in A549 cells.

### 3.6. The Effects of BA6 on the Protein Levels of Bcl-2 Family and the Intrinsic Pathway

**Figure 5:**
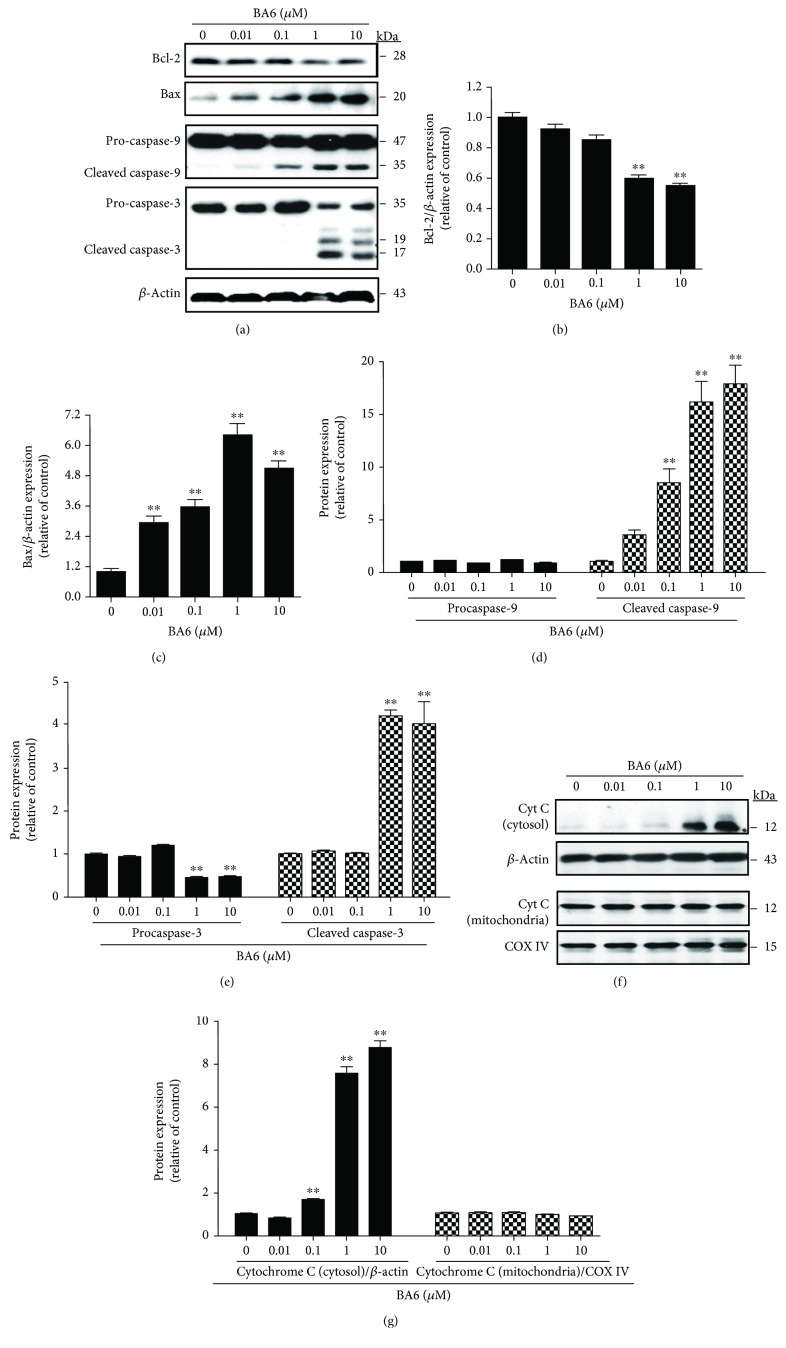
Effect of BA6 induced on the mitochondria-apoptotic (intrinsic) pathway protein expression in A549 cells. (a) A549 lung cancer cells were treated with the indicated doses of BA6 for 24 h. However, cell lysates and protein were loaded to Western blot analysis using antibodies including Bax, Bcl-2, caspase-9, caspase-3, and *β*-actin antibodies. Here, *β*-actin was detected as the internal control. The Bcl-2 (b), Bax (c), procaspase-9 and cleaved caspase-9 (d), and procaspase-3 and cleaved caspase-3 (e) protein levels were quantified by ImageJ software, normalized with that of the *β*-actin level, and expressed as a normalization of the control. (f) The release level of cytochrome C from the mitochondria to the cytosol during BA6-induced apoptosis was performed by Western blot analysis. After the indicated treatment for 24 h, mitochondrial/cytosolic proteins were separated using mitochondrial/cytosol isolation kits and then subjected to Western blot analysis with antibodies, including COX IV, cytochrome C, and *β*-actin. (g) Protein levels of cytosolic and mitochondrial cytochrome C were quantified by ImageJ software, normalized with that of the *β*-actin and COX IV levels, and expressed as a normalization of the control. The data (mean ± SEM) are representative of three technical replicates. Asterisks indicate statistically significant differences between the treated group and the untreated group; ^∗^ *p* < 0.05 and ^∗∗^ *p* < 0.01.

Programmed cell death, as initiated from the mitochondrial intermembrane under oxidative stress, is regulated by apoptosis-related proteins, including Bcl-2, Bax, and cytochrome C [18], while the downstream caspase-3 has been identified as the crucial executioner of apoptosis [19]. As shown in Figure 5(a), after the treatment of increasing doses of BA6 (0.01, 0.1, 1, and 10 *μ*M), the antiapoptotic Bcl-2 protein was downregulated by more than 40% with a concomitant 6.0-fold significant increase in the proapoptotic Bax protein expression under 1 and 10 *μ*M BA6 treatments (Figures 5(b) and 5(c)). Caspase signaling has been implicated in the induction of apoptosis and generally exists as an inactive proform enzyme in cells. The caspase cascade is triggered by extracellular ligands when cells face oxidative stress in microenvironments. According to our observations, BA6 treatment between 0.1-10 *μ*M induced cytochrome C release from the mitochondria to the cytosol, which was followed by the significant increase of the caspase-9-cleaved form (about 17.8-fold increment) and the caspase-3-cleaved form (about 4-fold increment), leading to apoptotic death (Figures 5(d) and 5(e)). To detect cytochrome C release from the mitochondria after BA6 treatment, A549 cells were treated with 0.01 to 10 *μ*M BA6 for 24 h, and then, cytosolic and mitochondria fractions were isolated for further Western blot analysis. As shown in Figures 5(f) and 5(g), BA6 treatment can induce rapid accumulation of cytoplasmic cytochrome C (about 8.7-fold increment) in a dose-dependent manner, with all protein normalization using *β*-actin. The cytochrome C of the mitochondria was not significantly changed, with protein normalization using COX IV (cytochrome C oxidase complex IV). These observations indicate that BA6 induced apoptosis in A549 cells via the regulation of Bcl-2 family, which results in cytochrome C being released from the mitochondria.

### 3.7. The Effect of Pretreatment with Mitochondria-Targeted Antioxidant (MitoTEMPO) on BA6-Induced mtROS Overexpression and MMP Dissipation

**Figure 6:**
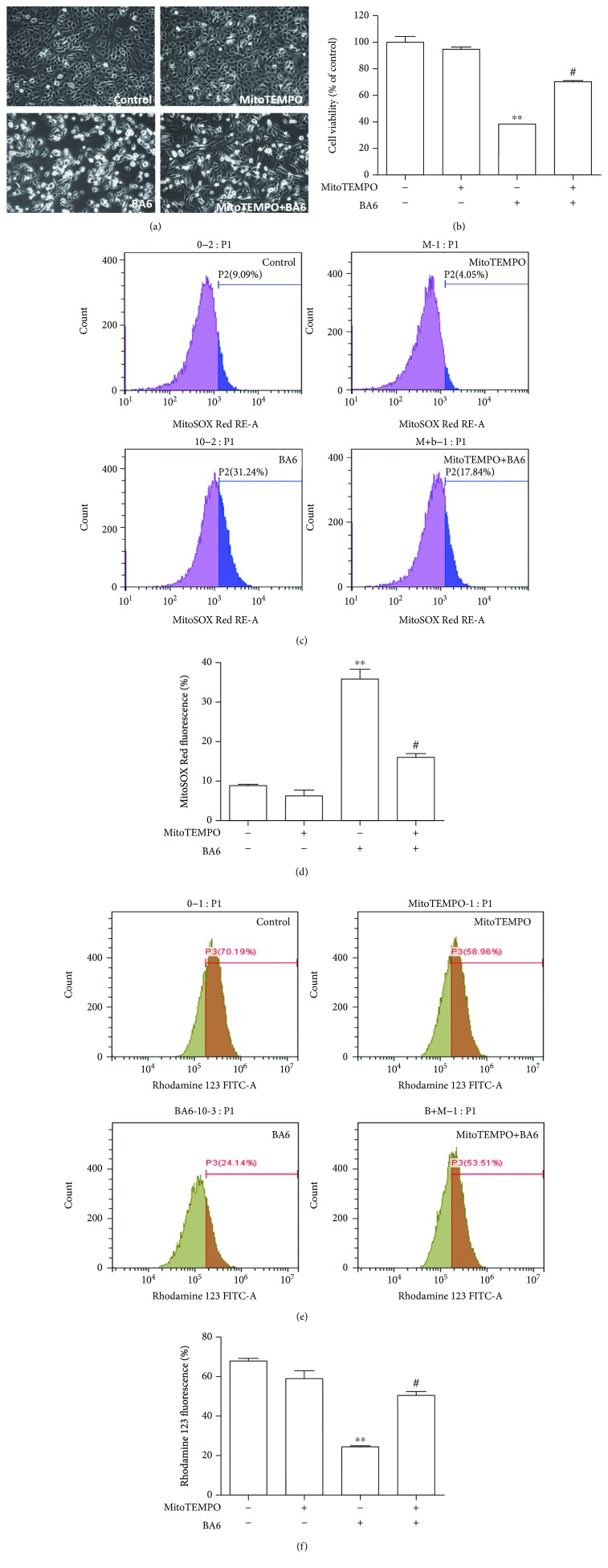
Effect of MitoTEMPO on the reverse of BA6-induced cellular cytotoxicity, mtROS production, and MMP disruption in the A549 cell. (a) After prior incubation with MitoTEMPO (10 *μ*M) for 4 h, A549 cells were treated without or with BA6 (10 *μ*M) for an additional 24 h and photographed by phase contrast microscopy at 200x magnification. (b) The proliferation of A549 cells was determined by the MTT assay, which is performed to measure cell viability. Cell viability (%) is expressed as a percentage, as compared to the untreated cells (0 *μ*M). The results are expressed as the mean ± SEM (*n* = 6) of three independent experiments. (c) The representative flow cytometry histogram demonstrates the effects of the MitoTEMPO on MitoSOX Red fluorescence expressions in A549 cells treated without or with BA6 (10 *μ*M) for 24 h. (d) Quantification of MitoTEMPO reversed the BA6-induced production of mtROS percentages by flow cytometry histogram. (e) The representative flow cytometry analysis demonstrates the effects of MitoTEMPO on rhodamine 123 fluorescence expressions in A549 cells treated without or with BA6 (10 *μ*M). (f) Quantification of inhibitory effects of MitoTEMPO on BA6-induced MMP disruption analyzed by rhodamine 123 and flow cytometry. Values are the mean ± SEM of three independent experiments. Significance was determined by Student's *t*-test: ^∗∗^ *p* < 0.01 and compared with untreated cells; ^#^ *p* < 0.05 as compared with the BA6 only group.

Our experimental results demonstrated that BA6 treatment increased mtROS production, destroyed MMP, and caused A549 cell apoptosis. MitoTEMPO, which is a mitochondria-specific antioxidant that eliminates excess mtROS [20], was applied to explore the role of mtROS in BA6-induced mitochondrial membrane disruption and apoptosis. We demonstrated that pretreatment of MitoTEMPO (10 *μ*M) for 4 h significantly reversed BA6-induced A549 cell apoptosis, as examined by phase contrast microscopy (200x magnification) and shown in Figure 6(a). Further MTT assay and statistics also proved that pretreatment with MitoTEMPO partially reversed the inhibitory effects of BA6 in A549 cell viability (Figure 6(b)). To verify whether MitoTEMPO inhibited the BA6-induced mtROS and MMP disruption, A549 cells were exposed with or without 10 *μ*M MitoTEMPO for 4 h and then incubated with or without BA6. Flow cytometry analysis was used to examine the changes of mtROS and integrity of MMP by MitoSOX Red and rhodamine 123 staining, respectively (Figures 6(c) and 6(e)). Our results reveal that MitoTEMPO partially reversed BA6-induced mtROS production and the disruption of MMP (Figures 6(d) and 6(f)). These data indicate that the induction of mtROS is required for BA6-induced cytotoxic effects and MMP disruption in A549 cells.

### 3.8. The Effect of Pretreatment with MitoTEMPO on BA6-Induced Apoptosis

**Figure 7:**
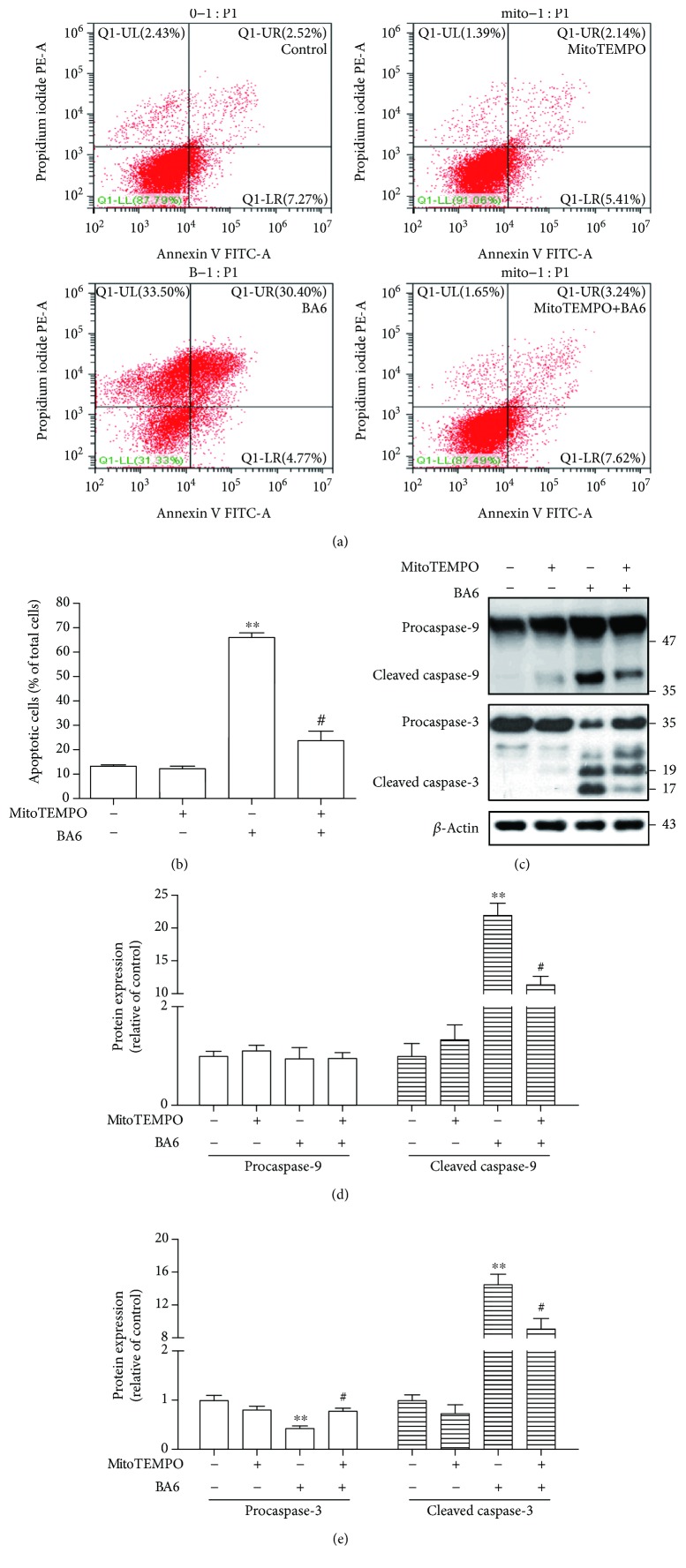
MitoTEMPO inhibited BA6-induced apoptosis in A549 cells. Pretreatment was conducted with MitoTEMPO (10 *μ*M) for 4 h, and then, A549 cells were treated without or with BA6 (10 *μ*M) for an additional 24 h. (a) This dot plot diagram was divided into four quadrants to present the ratio of apoptotic cells by using a flow cytometer. The representative flow cytometry analysis demonstrated the effects of MitoTEMPO on annexin V/PI staining expressions in A549 cells treated without or with BA6 (10 *μ*M). (b) The statistics show that MitoTEMPO is capable of reversing BA6-induced apoptosis (annexin V/PI double-positive and annexin V+/PI- staining). (c) The representative Western blot analysis demonstrated the effects of the MitoTEMPO on caspase-9 and caspase-3 protein expressions in A549 cells treated without or with BA6 (10 *μ*M). Here, *β*-actin was detected as the internal control. The procaspase-9 and cleaved caspase-9 (d) and procaspase-3 and cleaved caspase-3 (e) protein levels were quantified by ImageJ software, normalized with that of the *β*-actin level, and expressed as a normalization of the control. Values are the mean ± SEM of three independent experiments. Significance was determined by *t*-test: ^∗∗^ *p* < 0.01 as compared with untreated cells; ^#^ *p* < 0.05 as compared with the BA6 only group.

To determine whether MitoTEMPO attenuates BA6-induced cell apoptosis, A549 cells were treated with BA6, MitoTEMPO, or both for 24 h and then analyzed with annexin V/PI stain and flow cytometry. The percentage of apoptotic cells in the MitoTEMPO and BA6 cotreatment group was 23.6 ± 4.0%, as compared with the BA6 only group (66.0 ± 1.9%). MitoTEMPO significantly inhibits BA6-induced apoptosis in A549 cells (Figures 7(a) and 7(b)). The expression of cleaved caspase-9 was significantly decreased from the 22-fold change in the BA6 only group to an 11-fold change in the cotreatment group (Figures 7(c) and 7(d)). In addition, cotreatment with MitoTEMPO decreased the levels of cleaved caspase-3, including 17 and 19 kDa subunits, as compared with the BA6 only group (Figures 7(c) and 7(e)). Taken together, MitoTEMPO, the mitochondria-specific antioxidant, attenuates BA6-induced apoptosis and associated protein expression, emphasizing the importance of mtROS in apoptosis induction by BA6.

## 4. Discussion

Lung cancer has been the top three in cancer deaths worldwide in the past decade. Due to the lack of general and effective screening tools, most patients diagnosed with lung cancer are in the terminal stage [20]. Moreover, lung cancer cells easily develop resistance after chemotherapy. In the situation of a drug resistance problem, patients will be confronted with the predicament of no effective drugs being available [21]. In addition, distant metastasis usually occurs in lung cancer patients, as it commonly metastasizes to important organs, such as bones, brain, and liver. The metastasis often leads to impairment of organ function [22, 23]. Therefore, the development of new chemotherapeutic drugs is very important to improve the survival of lung cancer patients.

BA6, as isolated and purified from marine sponge, has the structure and molecular formula shown in Supplementary Figure 1. BA6 is a marine product with anticancer activity and has been found to inhibit human cancer cells, such as renal cancer cells [12], prostate cancer cells [13], cholangiocarcinoma cells [24], bladder cancer cells [25], and leukemia cells [26, 27]. This study demonstrated that BA6 can effectively reduce cell viability in lung cancer A549 cells (IC_50_ approximately 5.12 *μ*M), brain cancer GBM cells (IC_50_ approximately 7.12 *μ*M) and U87 cells (IC_50_ approximately 9.58 *μ*M), and hepatoma HepG2 cells (IC_50_ approximately 12.55 *μ*M) but does not inhibit cell viability in oral gingival cells (HGF-1) and oral mucosa cells (OMF). Our study shows the IC_50_ of BA6 in A549 cells is about 5 *μ*M, and this value is similar to prostate cancer cells found in the previous study [13]. BA6 drugs were found to have significant cytotoxicity in lung cancer cells at low concentrations of 1-10 *μ*M but had no effect on nontumor cells, which is the benefit of new drug development.

Most of the newly synthesized compounds with anticancer effects may have complex mechanisms, in which apoptosis-induced and regulation of the apoptosis signaling pathway is considered to play a key role [27]. First, we showed the effect of BA6 on A549 cell viability by MTT staining, followed by early and late apoptosis examined by annexin V/PI staining, and DNA fragmentation was through TUNEL stain analysis. Further, Western blot was used to detect procaspase-3 and cleaved caspase-3 expressions. The BA6-induced A549 cell apoptosis was proved by different methods in our study, and these results are similar to previous reports performed in various cancer cell lines [12, 13, 24, 25] but not reported in lung cancer cells.

Mammalian apoptosis has two important pathways of induction, extrinsic and intrinsic pathways [28, 29]. In the extrinsic pathway (Fas/FasL-mediated apoptosis), apoptosis is induced by binding the apoptosis signaling receptor (Fas-CD95) and Fas ligand (FasL), which further forms a death-inducing signaling complex (DISC) [30, 31]. On the other hand, the intrinsic pathway (mitochondria-mediated apoptosis) is dependent on the Bcl-2 family, whose members have the functions of proapoptotic proteins (Bid, Bax, Bak, etc.) and antiapoptotic proteins (Bcl-2, Bcl-xL, etc.). The Bcl-2 family also regulates the mitochondrial outer membrane permeabilization (MOMP), which will form pores on the mitochondrial membrane, and subsequently decrease mitochondrial membrane potential (Δ*ψ*m) and promote the release of cytochrome C from the mitochondria into the cytoplasm. Cytochrome C and caspase-9 combine to form apoptosome, and activates procaspase-3, which ultimately leads to cell apoptosis and death. This study demonstrates that the cytotoxic effect of BA6 in lung cancer cells is due to the damage of MOMP via the mitochondria-mediated apoptotic pathway and the release of cytochrome C from the mitochondria. This release appears to be considered a “point of no return” for apoptosis [32, 33]. The apoptosis process plays a key role in inhibiting drug resistance and tumor formation. Previous research reports and our study results all show that BA6 disrupted mitochondrial membrane potential and activated the intrinsic pathways that ultimately cause apoptosis [25, 26, 34]. Recently, many anticancer drug developments have focused on the modulation of this pathway for apoptosis [35].

The mitochondria are crucial for normal cell health and tumor cell survival [36] and is also a major site for the production of ROS. ROS plays important roles in the regulation of cellular physiology; a low concentration of ROS is necessary to maintain cell function, such as cell redox signaling; however, high concentration of ROS can be used as a mode of cytotoxicity and cause cell death [37]. Recent studies have indicated that the therapeutic effect of many chemotherapy drugs is realized due to the high concentrations of ROS production, which result in mitochondrial membrane damages and subsequent cancer cell apoptosis [37–39]. In order to prevent excessive ROS-induced cell damage and apoptosis, the cells and mitochondria contain cleansing ROS proteins (antioxidant enzymes) such as superoxide dismutase 1 (SOD1), superoxide dismutase 2 (SOD2), and catalase to reduce the toxicity of excessive ROS [40–42]. There is growing evidence that anticancer drugs that increase ROS can be used in cancer therapy [43]. Chen et al. reported that mtROS and total ROS were increased after BA6 treatment, but they did not evaluate the effects of BA6 on SOD1, SOD2, and catalase in their studies [26]. Therefore, to the best of our knowledge, our study is the first to demonstrate that BA6 effectively induced apoptosis in A549 cells through the production of both mtROS and intracellular ROS and the decrease of antioxidant enzymes, which ultimately leads to the destruction of the mitochondrial function.

The main function of mitochondria in cells is to provide energy for cell metabolism and biosynthesis through the production of adenosine 5′-triphosphate (ATP). Cells depend on two pathways to produce ATP, the OXPHOS reaction in the mitochondria and glycolysis in the cytoplasm. German scientist Otto Warburg believed that cancer cells use a large amount of glycolysis to produce energy in an aerobic environment because of defects in mitochondrial respiration [44]. However, current research suggests that the mitochondrial function is intact in most cancers; even though some cancers have higher glycolysis effects, they still maintain mitochondrial respiration [45, 46]. The tricarboxylic acid (TCA) cycle in the mitochondrial matrix utilizes the metabolic products of glucose to produce NADH, which it provides to the electron transport chains (ETC) situated at the mitochondrial inner membranes. Using the proton gradient produced from ETC as the driving force, mitochondrial ATP synthase catalyzes ADP phosphorylation to generate ATP, which is the carrier of cellular energy [47]. The MMP is the index of the mitochondrial functional integrity, and the potential integrity is mainly maintained by ETC and ATP synthase [48, 49]. We used extracellular flux analysis to measure mitochondrial respiratory and bioenergetic activity in cells and recorded the oxygen consumption rate (OCR) and extracellular acidification rate (ECAR), which further shows the mitochondrial respiration (oxidative phosphorylation) and glycolysis. Among them, the extracellular OCR can understand the basal respiratory rate, ATP production rate, proton leak rate, maximum respiratory rate, and spare respiratory capacity [10, 50]. Our study demonstrated that BA6 reduces glycolysis and oxidative phosphorylation activity (including basal respiration rate, ATP production rate, maximum respiratory rate, and spare respiration capacity) in lung cancer cells but does not affect the proton leakage rate. We also confirmed that BA6 treatment disrupts mitochondrial membrane potential and significantly inhibits ATP5A protein expression and ATP production. The underlying mechanism of BA6-induced apoptosis is through the inhibition of mitochondrial oxidative phosphorylation and ATP production in A549 cells. Hence, this is the first paper to demonstrate that BA6 has the capacity of inhibiting mitochondrial OXPHOS and glycolysis in lung cancer cells.

MitoTEMPO is a mitochondria-targeted antioxidant that protects complexes I, II, and III in the electronic respiratory chain, restores MnSOD activity, and reduces ROS production [51]. MitoTEMPO is approved to maintain mitochondrial integrity and reduce ATP depletion recovery-mediated necrosis and apoptosis [52]. *In vitro* studies of MitoTEMPO have indicated its protective effect on mitochondrial dysfunction and mitochondria-mediated oxidative stress [53]. Our study demonstrated that BA6 reduces lung cancer cell survival by increasing mtROS production, modulating Bcl-2 family proteins, and destroying the mitochondrial membrane potential. The pretreatment with MitoTEMPO significantly reduced the BA6-induced cytotoxicity, ROS overproduction, and MMP disruption and finally inhibited the caspase-9 and caspase-3 activation and reduced apoptosis (early and late apoptosis) induction in lung cancer cells. Our results demonstrated that BA6 induced excessive ROS production and mitochondrial dysfunction, while exogenous MitoTEMPO can effectively reduce the BA6-induced apoptosis and related pathways in A549 cells. Many anticancer drugs commonly used in the treatment of lung cancer, such as gemcitabine and paclitaxel, have been shown to produce anticancer effects through increasing intracellular oxidative stress. Our study showed that BA6 possessed similar abilities as the above-mentioned chemotherapeutic drugs, as well as a series of downstream reactions to induce cancer cell apoptosis.

## 5. Conclusion

**Figure 8:**
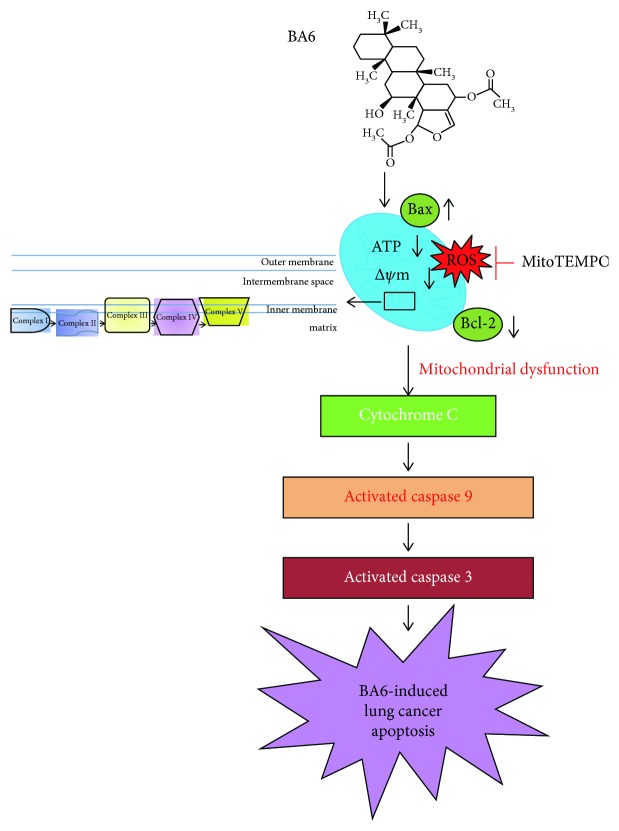
The scheme for the involvement of the mitochondrial apoptosis pathway in BA6-induced cytotoxicity in A549 cells. BA6 causes ROS accumulation, depolarization of mitochondrial membrane potential, exhaustion of ATP, inhibition of OXPHOS, and increase of cytochrome C release to cytoplasm, which results in caspase activation towards the intrinsic (mitochondria-mediated) apoptosis pathway.

In view of the results of this study, we summarized the signaling pathway based on the BA6-induced apoptosis mechanism in human lung cancer A549 cells (Figure 8). Firstly, BA6 increased the production of mtROS and regulated the Bcl-2 family, including upregulating the expression of proapoptotic protein Bax and downregulating the expression of antiapoptotic protein Bcl-2, which damages MOMP. On the other hand, the oxidative phosphorylation for the biological activity of the mitochondrial inner membrane was reduced, which ultimately led to the decrease in the MMP (Δ*ψ*m) and reduction of ATP production in the mitochondria. Disruption of MMP promotes the release of cytochrome C from the mitochondria into the cytoplasm, stimulates the activation of caspase-9 and caspase-3, and finally, leads to apoptosis of lung cancer cells. Induction of the intrinsic (mitochondria-mediated) apoptotic pathway is the underlying mechanism of BA6-induced apoptosis. Pretreatment with mitochondria-targeted antioxidant (MitoTEMPO) could effectively reduce the BA6-mediated apoptosis and related protein expressions. These results indicate that BA6 induces apoptosis by increasing mtROS and inducing mitochondrial dysfunction. Our study demonstrates that BA6, as a promising chemotherapeutic compound, induces excessive oxidative stress, thereby, further inducing apoptosis of lung cancer cells. The above features indicate that BA6 has the potential to be researched and developed into clinical drugs for the treatment of lung cancer.

## Data Availability

The testing compound and experimental data used to support the findings of this study are included within the article and the supplementary information file.

## Supplementary Materials

Supplementary 1 Figure 1: chemical molecular structure of BA6: empirical formula is C_29_H_44_O_6_, and molecular weight is 488.66.

Supplementary 2 Movie 01: apoptosis of A549 cells treated with BA6 was detected by living cell tomographic microscopy. A549 cells were seeded in a glass bottom microwell dish overnight; shooting started immediately after 10 *μ*M BA6 injection. Photo was taken every 3 minutes; shooting time is for 3 hours. The upper left and upper right cells are detached immediately after drug injection, and the filamentous cell membrane is slowly removed from the bottom of the cell disk. Two cells in the middle immediately produce a large number of apoptotic bodies in 30 min after drug injection.
