# Supplementary figures and images for: BA6 Induces Apoptosis via Stimulation of Reactive Oxygen Species and Inhibition of Oxidative Phosphorylation in Human Lung Cancer Cells
Source: Oxid Med Cell Longev. 2019 May 7;2019:6342104. doi: 10.1155/2019/6342104 (PMC6530211; doi:10.1155/2019/6342104)

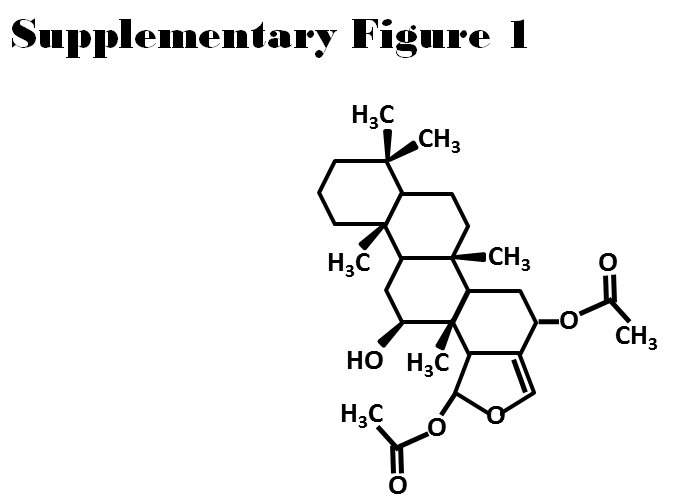

Supplement: Supplementary 1 — Figure 1: chemical molecular structure of BA6: empirical formula is C29H44O6, and molecular weight is 488.66. [file 6342104.f1.TIF]
